# The effect of resveratrol on the cryopreservation of Mongolian horse semen

**DOI:** 10.5194/aab-68-27-2025

**Published:** 2025-01-06

**Authors:** Ming Du, Yuanyi Liu, Lei Zhang, Xinyu Li, Na Wang, Qianqian He, Jialong Cao, Bilig Zhao, Yujie Shi, Bei Li, Gerelchimeg Bou, Manglai Dugarjaviin

**Affiliations:** 1 Key Laboratory of Equus Germplasm Innovation, Ministry of Agriculture and Rural Affairs, Hohhot 010018, China; 2 Inner Mongolia Key Laboratory of Equine Science Research and Technology Innovation, Inner Mongolia Agricultural University, Hohhot 010018, China; 3 Equus Research Center, Inner Mongolia Agricultural University, Hohhot 010018, China

## Abstract

Cryopreservation of semen has advanced significantly with the development of artificial insemination techniques, but post-thawed sperm often exhibit reduced viability, membrane integrity, and acrosome integrity compared to fresh sperm, leading to decreased fertilization capacity. Oxidative stress is a major concern during cryopreservation. This study investigated the use of resveratrol (RSV), a potent antioxidant, in the cryopreservation of Mongolian horse semen. Different concentrations of RSV were incorporated into semen cryopreservation extenders, and the morphological and antioxidant indices of post-thawed sperm were assessed to determine the optimal RSV concentration. The study also employed tandem mass tag (TMT) quantitative proteomics technology to explore differential proteins and their pathways. The results showed that sperm quality parameters were positively correlated with RSV concentration within a certain range (10–40 µ mol L^−1^) and were significantly higher than the control group. RSV also enhanced the antioxidant capacity of sperm, with the optimal effect observed at 40 µ mol L^−1^. Proteomics analysis identified 10 differential proteins between the control and optimal RSV concentration groups, with 7 upregulated proteins primarily involved in antioxidant activity and maintaining intracellular redox balance. These findings were further validated through real-time fluorescent quantitative PCR and protein immunoblotting, suggesting that RSV has potential as an effective antioxidant for improving the cryopreservation of Mongolian horse semen.

## 1 Introduction

Semen cryopreservation has emerged as a crucial technique in modern animal husbandry, enabling the long-term preservation of genetic resources and enhancing the efficiency of artificial insemination (Yang et al., 2020; Rusco et al., 2022; Takeo et al., 2022). However, despite significant advances, post-thawed sperm often exhibit reduced quality in terms of motility, membrane integrity, and fertilizing capacity compared to fresh sperm. This decline in sperm quality is attributed to various factors, including oxidative stress and ice crystal formation during the freezing and thawing processes (Jakop et al., 2023).

Previous studies have shown that the addition of antioxidants to semen diluents can mitigate the oxidative damage caused by reactive oxygen species (ROSs) during cryopreservation (Li et al., 2023). Among these antioxidants, resveratrol (RSV) has garnered considerable attention due to its potent antioxidant and pleiotropic biological activities. RSV has been reported to enhance sperm quality in various species through its ability to scavenge free radicals and maintain redox homeostasis (Garcez et al., 2010; Rezaie et al., 2021). However, the specific mechanisms underlying RSV's protective effects on cryopreserved sperm, particularly in the context of Mongolian horses, remain largely unexplored.

Mongolian horses are an important breed with unique genetic characteristics and superior performance abilities (Du et al., 2024). However, their geographical distribution and limited population size pose challenges for genetic improvement and conservation (Liu et al., 2024). Therefore, optimizing semen cryopreservation techniques for Mongolian horses is crucial for safeguarding their genetic diversity and enhancing their reproductive potential.

The present study aims to evaluate the effects of RSV on the cryopreservation of Mongolian horse semen. Specifically, we investigated the optimal concentration of RSV in semen diluents required to maximize sperm quality parameters such as motility, membrane integrity, and antioxidant capacity. Furthermore, we employed tandem mass tag (TMT) quantitative proteomics to identify differentially expressed proteins between control and RSV-treated samples, providing insights into the molecular mechanisms underlying RSV's protective effects. Our findings will contribute to the development of improved semen cryopreservation protocols for Mongolian horses and potentially for other equine species.

## 2 Materials and methods

### 2.1 Semen sample collection

The equine semen samples used in this study were collected from six healthy Mongolian horses housed at the White Horse Breeding Base in Xiwuzhumuqin Banner, Xilin Gol League, Inner Mongolia Autonomous Region, China. The ejaculates, appearing milky white in color, underwent rigorous screening, with only those having sperm motility of 80 % or higher and a sperm density of no less than 1.0 × 10^9^ sperms mL^−1^ being selected for subsequent experimental analyses. The semen of six horses will be mixed for the subsequent measurement of relevant indicators. All sampling procedures were approved by the Laboratory Animal Welfare and Ethics Committee of Inner Mongolia Agricultural University and complied with the required standards.

### 2.2 Preparation of equine semen extender

For the preparation of the base solution, combine the following reagents in a sterile container: 5.0 g of glucose, 0.3 g of lactose, 0.3 g of raffinose, 0.06 g of sodium citrate, 0.082 g of potassium citrate, and 0.476 g of HEPES (4-hydroxyethyl piperazine ethanesulfonic acid), all sourced from Sigma, USA; 10 000 IU of penicillin from Jinhua Conba Pharmaceutical Co., Ltd., Zhejiang, China; and 1.0 mg of gentamicin from Boao Tuoda Technology Co., Ltd., Beijing, China. Add double-distilled water to dissolve the ingredients, adjust the volume to 100 mL, and mix thoroughly with a magnetic stirrer at room temperature for 30 min. To prepare the skim milk solution, follow the package instructions to dissolve 10.0 g of skim milk powder in double-distilled water to make 100 mL, mixing thoroughly for 10–20 min using a magnetic stirrer at room temperature. Fresh egg yolks are obtained by separating the yolks from the whites and piercing the yolk membrane. Diluent I is composed of 50 mL of the base solution and 50 mL of the skim milk solution, along with 5 % yolk, and is kept in a 37°C water bath for no longer than 2 h. Diluent II, or the freezing extender, consists of 50 mL of the base solutions and 50 mL of the skim milk solution, 5 % yolk, and 5 % glycerol (also from Sigma, USA) and should be kept at room temperature for no more than 2 h (He et al., 2020).

### 2.3 Semen freezing and thawing

Dilute the semen using the prepared freezing extender and divide it into nine equal groups. To ensure RSV concentrations of 0, 10, 20, 30, 40, 50, 60, 80, and 100 µ mol L^−1^ in each group, calculate and add precise amounts of RSV stock solution to each semen sample and then mix thoroughly for uniformity. Adjust the sperm density in each sample to 5 × 10^7^ sperms mL^−1^. Dispense the semen into 0.5 mL straws and equilibrate at 4°C for 100 min. Subsequently, expose the straws to vapor at 3 cm above liquid nitrogen for 15 min and then store them in liquid nitrogen for long-term preservation. After 30 d, thaw the samples by immersing them in a 37°C water bath for 30 s. For each group, select three straws for thawing, and analyze the semen samples within 15 min after thawing to ensure data accuracy and reliability (Almeida et al., 2017; Elkhawagah et al., 2020).

### 2.4 Sperm motility detection

Measure sperm motility using a portable sperm motility analyzer (AndroScope, Minitube GmbH, Germany), which calculates motility as the proportion of straight-moving sperms to the total sperm count. The procedure involves placing 10 µ L of semen sample in a Leja 20 mm four-chamber slide and then measuring sperm motility at 37°C. To ensure data accuracy and reliability, randomly select five different fields of view in the chamber, observe at least 200 sperms in each field, and repeat the test three times for each semen sample.

### 2.5 Detection of frozen semen acrosome integrity

Assess acrosome integrity using FITC-PNA fluorescent dye (Thermo Fisher Scientific Inc., Waltham, MA, USA) . Green fluorescence indicates acrosome damage. Dilute the thawed semen with phosphate buffer saline (PBS) at a 1:1 ratio. Centrifuge at 650 g for 7 min, remove the supernatant, wash with 1 mL of PBS, and repeat this process three times. Add 20 µ L of FITC-PNA solution and incubate at 36° for 10 min. Observe the samples under a fluorescence microscope. For data accuracy and reliability, thaw three straws per group, randomly select five different fields of view from each straw, count at least 200 sperms per sample, and calculate the acrosome integrity rate based on the observed fluorescence (Inanç et al., 2022).

### 2.6 Detection of frozen semen plasma membrane integrity

Detect plasma membrane integrity using the SYBR-14/PI detection kit (Thermo Fisher Scientific Inc., Waltham, MA, USA) . Orange fluorescence on the entire sperm head indicates membrane damage. Incubate 1 mL of semen in a 1.5 mL centrifuge tube at 37°C for 30 min, centrifuge at 600 g for 5 min, carefully remove the supernatant, add 1 mL of dye solution A, mix well, count using a hemocytometer, and adjust to 2 × 10^7^ sperm cells mL^−1^. Transfer 100 µ L of sperm cells to a new 1.5 mL centrifuge tube, add 10 µ L of dye solution A, mix, incubate for 10 min (avoid light), add dye solution B, incubate for another 10 min (avoid light), then transfer 50 µ L to a glass slide, carefully remove the dye solution, cover with a coverslip, and immediately observe and count under a fluorescence microscope. For each group, thaw three straws, randomly select five observation fields from each straw, ensure at least 200 sperms per sample, and calculate the plasma membrane integrity rate (Olexikova et al., 2019; Zou et al., 2022).

### 2.7 Measurement of total antioxidant capacity (T-AOC), superoxide dismutase (SOD), malondialdehyde (MDA) content, and glutathione peroxidase (GSH-Px) activity

Measure total antioxidant capacity (T-AOC) (BC1310, Solarbio Science & Technology Co., Ltd., Beijing, China), superoxide dismutase (SOD) (BC0170, Solarbio Science & Technology Co., Ltd., Beijing, China), malondialdehyde (MDA) content (BC0025, Solarbio Science & Technology Co., Ltd., Beijing, China), and glutathione peroxidase (GSH-Px) (EEA010, Thermo Fisher Scientific Inc., Waltham, MA, USA) activity strictly according to the instructions provided in the corresponding kits (Lu et al., 2015; Colakoglu et al., 2017; Tsikas, 2017; Nazif et al., 2022) . The detailed results of the relevant indicators can be found in Table S1 in the Supplement.

### 2.8 Proteomics sequencing

Perform quantitative analysis using proteome discovery software, which employs the isobaric labeling method based on unique peptide intensity summation for protein quantification. Protein intensity is calculated by summing the intensities of its matched unique peptides. After obtaining protein quantitative data, further normalize the protein quantitative data by extracting protein intensities from different samples and applying central transformation to obtain relative quantitative values ( R ). Then, perform differential quantitative analysis to identify differentially expressed proteins among different sample groups. Use the t test to calculate fold change (FC) and the corresponding P value, ensuring the accuracy and reliability of protein quantitative data for subsequent biological analysis (Jia et al., 2021).

### 2.9 Primer design and synthesis

The gene sequences of *HSPA6*, *PRDX5*, *GPX4*, *CD47*, and *PDIA6* in horses were retrieved from the NCBI website (https://www.ncbi.nlm.nih.gov, accessed on 21 January 2024). Specific primers were designed using Premier (Table S2), and the beta-actin ( β -*actin*) gene was selected as the internal reference gene. The primers were synthesized by Sangon Biotech (Shanghai) Co., Ltd (Wang et al., 2021).

### 2.10 Real-time fluorescence quantitative PCR

Real-time quantitative PCR using a fluorescent quantitative PCR detection system (Bio-Rad, Hercules, California, USA). The relative gene expression levels were calculated using the 2^−ΔΔCt^ method (Table S3) (Han et al., 2022).

### 2.11 Western blot

Proteins were extracted from the samples using an extraction buffer. Subsequently, the total protein content in the extraction buffer was measured using a quantitative method. The extraction buffer was electrophoresed to separate the proteins, which were then blotted onto a specific membrane. The proteins on the blotted membrane were eluted with an elution buffer to eliminate other nonspecific proteins. Finally, the eluted proteins were subjected to immunodetection (Mishra et al., 2017; Xing et al., 2023a). The information related to antibodies is shown in Table S4.

### 2.12 Statistical model

All data from this experiment were analyzed using SPSS software version 22.0 for one-way ANOVA analysis of variance. All results are expressed as mean ± standard deviation (mean ± SD). P<0.05 indicates a significant difference, while P>0.05 indicates no significant difference (Kruppa and Hothorn, 2020; Ren et al., 2022).

**Table 1:** Effects of different concentrations of RSV on sperm motility of horse semen after freezing.

Number	Group	Numbers ( n )	Sperm motility rate (%)
1	Fresh semen	3	94.49 ± 1.01^a^
2	0 µ mol L^−1^	3	24.86 ± 2.23^f^
3	10 µ mol L^−1^	3	30.28 ± 2.94^e^
4	20 µ mol L^−1^	3	34.00 ± 2.43^cd^
5	30 µ mol L^−1^	3	35.33 ± 1.12^c^
6	40 µ mol L^−1^	3	44.64 ± 1.28^b^
7	50 µ mol L^−1^	3	32.26 ± 1.35^de^
8	60 µ mol L^−1^	3	30.78 ± 1.15^e^
9	80 µ mol L^−1^	3	27.10 ± 1.70^f^
10	100 µ mol L^−1^	3	26.72 ± 0.61^f^

Note that different lowercase letters on the shoulder label of the same column indicate significant differences ( P<0.05 ); the same lowercase letters on the shoulder label indicate no significant difference ( P>0.05 ). The same applies to the following tables.

## 3 Results

### 3.1 Effect of resveratrol on morphological indices of horse sperm

#### 3.1.1 Effect of different concentrations of resveratrol on the viability of sperm after freezing Mongolian horse semen 

As indicated in Table 1, the viability of sperm in fresh semen was significantly higher than that in frozen semen ( P<0.05 ). Within the concentration range of 0 to 40 µ mol L^−1^, the sperm viability increased correspondingly with the gradual increase in RSV concentration. However, when the RSV concentration exceeded a certain level, its protective effect on sperm decreased significantly. When the RSV concentration reached 40 µ mol L^−1^, the viability of frozen sperm was the highest, reaching 44.64 %, which was significantly higher than that of the control group and other experimental treatment groups ( P<0.05 ).

#### 3.1.2 Effect of different concentrations of resveratrol on the acrosome integrity rate of sperm after freezing Mongolian horse semen 

As can be seen from Table 2, with the increase in RSV concentration, the acrosome integrity rate of horse semen after freezing showed a trend of increasing first and then decreasing. When the RSV concentration reached 40 µ mol L^−1^, the acrosome integrity rate of Mongolian horse sperm was significantly higher than that of the control group and other experimental treatment groups, reaching 74.97 % ( P<0.05 ).

**Table 2:** Effects of different concentrations of RSV on the acrosome integrity rate of horse semen after freezing.

Number	Group	Numbers ( n )	Acrosome integrity rate (%)
1	0 µ mol L^−1^	3	51.68 ± 0.93^f^
2	10 µ mol L^−1^	3	62.24 ± 1.32^d^
3	20 µ mol L^−1^	3	67.39 ± 1.44^bc^
4	30 μ mol L^−1^	3	69.61 ± 0.44^b^
5	40 µ mol L^−1^	3	74.97 ± 3.16^a^
6	50 µ mol L^−1^	3	66.69 ± 0.93^c^
7	60 µ mol L^−1^	3	62.29 ± 0.96^d^
8	80 µ mol L^−1^	3	60.30 ± 0.97^d^
9	100 µ mol L^−1^	3	57.29 ± 1.45^e^

#### 3.1.3 Effects of different concentrations of resveratrol on the plasma membrane integrity of post-frozen Mongolian horse semen 

As can be seen from Table 3, within the concentration range of 10 to 40 µ mol L^−1^, the plasma membrane integrity rate after freezing increased significantly with the increase in RSV concentration ( P<0.05 ). However, when the RSV concentration exceeded 40 µ mol L^−1^, its protective effect on the integrity of the sperm plasma membrane decreased significantly. When 40 µ mol L^−1^ of RSV was added, the plasma membrane integrity rate of frozen horse sperm was significantly higher than that of the control group and other experimental treatment groups, reaching 55.69 % ( P<0.05 ).

**Table 3:** Effects of different concentrations of RSV on the plasma membrane integrity rate of horse semen after freezing.

Number	Group	Numbers ( n )	Plasma membrane integrity (%)
1	0 µ mol L^−1^	3	42.07 ± 1.28^h^
2	10 µ mol L^−1^	3	45.63 ± 0.64^ef^
3	20 µ mol L^−1^	3	48.30 ± 0.49^c^
4	30 µ mol L^−1^	3	50.28 ± 1.39^b^
5	40 µ mol L^−1^	3	55.69 ± 0.68^a^
6	50 µ mol L^−1^	3	47.99 ± 0.10^cd^
7	60 µ mol L^−1^	3	46.91 ± 0.25^de^
8	80 µ mol L^−1^	3	45.09 ± 0.73^fg^
9	100 µ mol L^−1^	3	43.92 ± 0.27^g^

### 3.2 Antioxidant role of resveratrol in the cryopreservation of Mongolian horse semen

#### 3.2.1 Effect of different concentrations of resveratrol on the total antioxidant capacity (T-AOC) of horse semen after freezing 

As shown in Table 4, the total antioxidant capacity (T-AOC) of horse semen after freezing increased initially and then decreased with the increase in RSV concentration. Within the concentration range of 10 to 50 µ mol L^−1^, T-AOC remained at relatively high levels, although the differences were not statistically significant ( P>0.05 ). However, the mean value in the 40 µ mol L^−1^ group was higher than that in other groups. Nevertheless, when the concentration exceeded 60 µ mol L^−1^, T-AOC gradually decreased, suggesting that high concentrations of RSV might have a negative impact on antioxidant capacity.

**Table 4:** Effects of different concentrations of RSV on total antioxidant capacity (T-AOC) of horse semen after freezing.

Number	Group	Numbers ( n )	T-AOC (U mL^−1^)
1	0 µ mol L^−1^	3	0.86 ± 0.65^b^
2	10 µ mol L^−1^	3	2.71 ± 0.43^a^
3	20 µ mol L^−1^	3	2.71 ± 0.77^a^
4	30 µ mol L^−1^	3	2.80 ± 0.36^a^
5	40 µ mol L^−1^	3	2.80 ± 0.56^a^
6	50 µ mol L^−1^	3	2.55 ± 1.00^a^
7	60 µ mol L^−1^	3	1.97 ± 0.44^a^
8	80 µ mol L^−1^	3	1.89 ± 0.70^ab^
9	100 µ mol L^−1^	3	1.77 ± 0.38^ab^

#### 3.2.2 Effect of different concentrations of resveratrol on the malondialdehyde (MDA) content in horse semen after freezing

As presented in Table 5, the malondialdehyde (MDA) content in horse semen after freezing initially decreased and then increased with the increase in resveratrol (RSV) concentration. Specifically, at a concentration of 40 µ mol L^−1^, the MDA content was significantly lower than that of other groups ( P<0.05 ).

**Table 5:** Effects of different concentrations of RSV on the content of malondialdehyde (MDA) in horse semen after freezing.

Number	Group	Numbers ( n )	MDA (nmol mL^−1^)
1	0 µ mol L^−1^	3	13.33 ± 1.04^a^
2	10 µ mol L^−1^	3	7.17 ± 0.29^d^
3	20 µ mol L^−1^	3	7.50 ± 0.50^d^
4	30 µ mol L^−1^	3	7.00 ± 0.50^d^
5	40 µ mol L^−1^	3	5.50 ± 1.00^e^
6	50 µ mol L^−1^	3	7.50 ± 0.50^d^
7	60 µ mol L^−1^	3	8.17 ± 1.04^cd^
8	80 µ mol L^−1^	3	9.33 ± 0.58^c^
9	100 µ mol L^−1^	3	10.67 ± 0.76^b^

#### 3.2.3 Effect of different concentrations of resveratrol on the activity of superoxide dismutase (SOD) in horse semen after freezing 

Table 6 presents the effect of different concentrations of RSV on the activity of superoxide dismutase (SOD) in horse semen after freezing. The data in the table indicate that, with the increase in RSV concentration, the SOD activity in horse semen after freezing showed a trend of increasing initially and then decreasing. Specifically, at a concentration of 40 µ mol L^−1^, the SOD activity was significantly higher than that of other groups ( P<0.05 ).

**Table 6:** Effects of different concentrations of RSV on the activity of superoxide dismutase (SOD) in horse semen after freezing.

Number	Group	Numbers ( n )	SOD (U mL^−1^)
1	0 µ mol L^−1^	3	14.22 ± 0.19^e^
2	10 µ mol L^−1^	3	14.89 ± 0.17^d^
3	20 µ mol L^−1^	3	15.73 ± 0.12^c^
4	30 µ mol L^−1^	3	16.07 ± 0.08^b^
5	40 µ mol L^−1^	3	16.27 ± 0.09^a^
6	50 µ mol L^−1^	3	15.55 ± 0.08^c^
7	60 µ mol L^−1^	3	14.73 ± 0.10^d^
8	80 µ mol L^−1^	3	14.82 ± 0.08^d^
9	100 µ mol L^−1^	3	14.73 ± 0.04^d^

#### 3.2.4 Effect of different concentrations of resveratrol on the activity of glutathione peroxidase (GSH-Px) in horse semen after freezing 

Table 7 presents the impact of different concentrations of RSV on the activity of glutathione peroxidase (GSH-Px) in horse semen after freezing. The data in the table reveal that, as the RSV concentration increased, the GSH-Px activity in horse semen after freezing exhibited a trend of initially increasing and then decreasing. Specifically, at a concentration of 40 µ mol L^−1^, the GSH-Px activity was significantly higher than that of the other groups ( P<0.05 ).

**Table 7:** Effects of different concentrations of RSV on the activity of glutathione peroxidase (GSH-Px) in horse semen after freezing.

Number	Group	Numbers ( n )	GSH-Px ( µ mol mL^−1^)
1	0 µ mol L^−1^	3	53.86 ± 10.36^d^
2	10 µ mol L^−1^	3	83.09 ± 16.14^c^
3	20 µ mol L^−1^	3	95.03 ± 11.38^bc^
4	30 µ mol L^−1^	3	112.40 ± 20.38^b^
5	40 µ mol L^−1^	3	153.55 ± 24.18^a^
6	50 µ mol L^−1^	3	95.07 ± 12.02^bc^
7	60 µ mol L^−1^	3	89.08 ± 8.32^bc^
8	80 µ mol L^−1^	3	70.47 ± 8.08^cd^
9	100 µ mol L^−1^	3	73.12 ± 13.61^cd^

### 3.3 Differential proteins between the control group and optimal resveratrol concentration group of Mongolian horse spermatozoa

#### 3.3.1 Analysis of differential proteins in spermatozoa 

**Figure 1:**
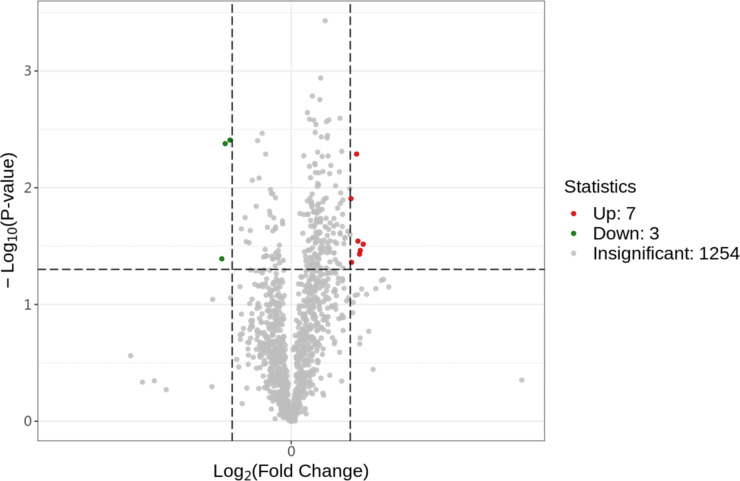
Differential protein volcano plot; the abscissa represents the change in protein expression fold change, and the ordinate represents the significance level of differential protein abundance.

After searching, a total of 1254 proteins were identified in the UniProt Equus 137820 20231029.fasta database. Proteins with a fold change (FC) of ≥ 1.3 or ≤ 0.7692 and a P value of ≤ 0.05 were considered to be differentially abundant proteins (DAPs). A volcano plot was generated to identify DAPs (Fig. 1). Compared to the control group (0 µ mol L^−1^), the treatment group (40 µ mol L^−1^) exhibited seven upregulated proteins (HSPA6, PRDX5, GPX4, PIK3C2B, COX2, DNAI4, KLK1E2) and three downregulated proteins (PDIA6, CD47, IDS).

**Figure 2:**
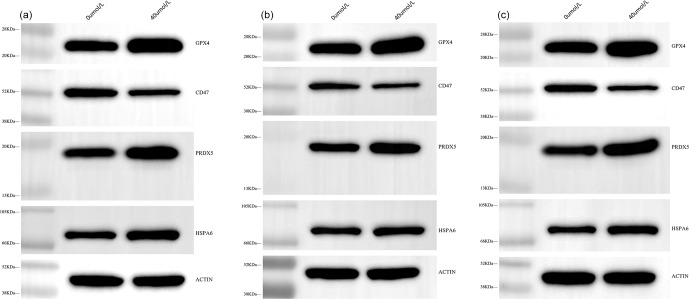
WB bands of HSPA6, PRDX5, GPX4, CD47, and β -actin proteins. Panels **(a)**, **(b)** and **(c)** are the three repeated experiments.

**Figure 3:**
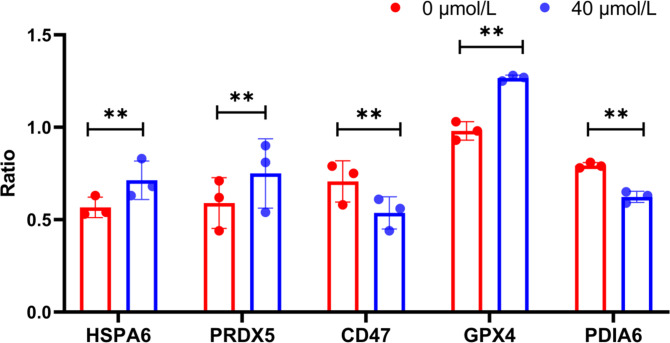
WB index gray value / internal reference gray value (ratio) of HSPA6, PRDX5, GPX4, PDIA6, and CD47 proteins; ^**^ indicates P<0.01 .

Furthermore, the expression levels of HSPA6, PRDX5, GPX4, PDIA6, and CD47 at the protein level were investigated using western blotting (WB) (Fig. 2). The results were highly consistent with the previous sequencing data. Specifically, the expression levels of HSPA6, PRDX5, and GPX4 in the treatment group were significantly higher than those in the control group ( P<0.05 ), while the expression levels of CD47 were significantly lower ( P<0.05 ) (Fig. 3). These results further confirmed the accuracy of the sequencing data.

**Figure 4:**
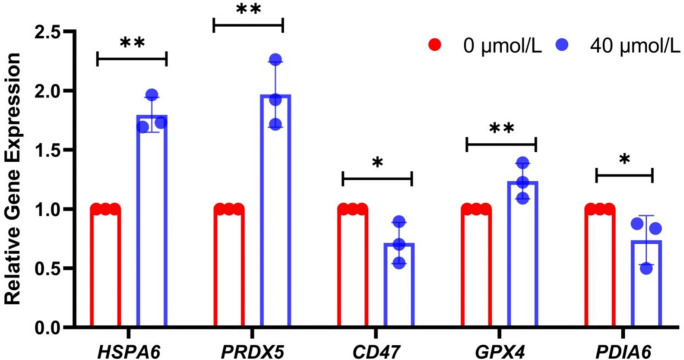
Verification of sequencing results by real-time fluorescence quantitative PCR; ^*^ indicates P<0.05 , and ^**^ P<0.01 .

Additionally, to further validate the authenticity of these differentially expressed proteins, real-time fluorescent quantitative PCR experiments were conducted. The results showed that the relative expression levels of *HSPA6*, *PRDX5*, and *GPX4* in the treatment group were significantly higher than those in the control group ( P<0.05 ) (Fig. 4), while the relative expression levels of *PDIA6* and *CD47* were significantly lower ( P<0.05 ). These results further corroborated the accuracy of the sequencing data and provided a foundation for further exploring the molecular mechanisms underlying the low-temperature tolerance of Mongolian horse spermatozoa.

#### 3.3.2 Differential protein enrichment analysis 

**Figure 5:**
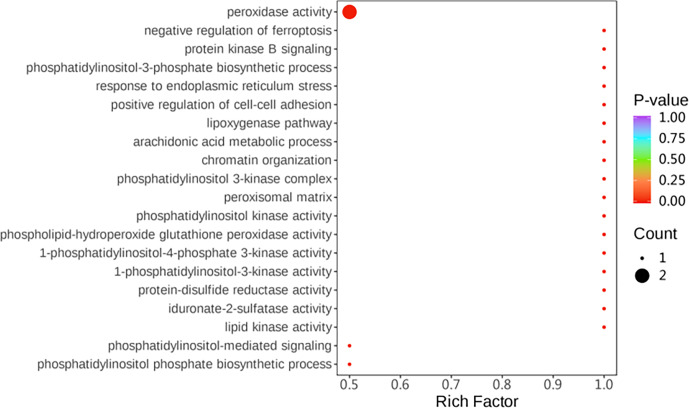
GO functional enrichment analysis of highly expressed genes.

Gene ontology (GO) enrichment analysis was used to investigate the differential proteins between two groups. The differential proteins were mainly enriched with regard to the following: peroxidase activity, response to endoplasmic reticulum stress, and phospholipid-hydroperoxide glutathione peroxidase activity (Fig. 5).

**Figure 6:**
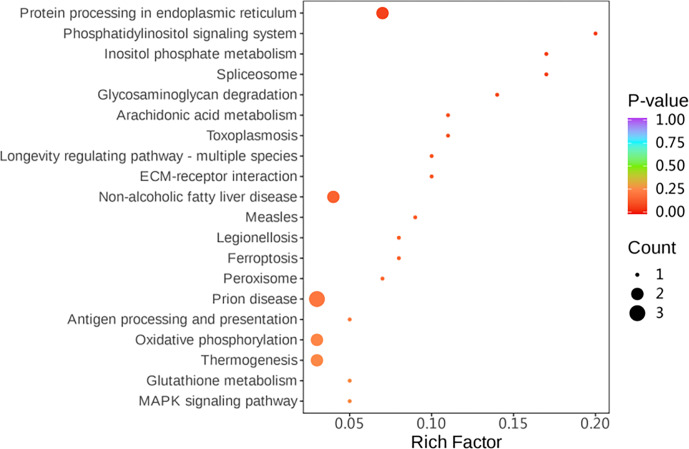
KEGG signalling pathway of highly expressed genes.

Simultaneously, KEGG (Kyoto Encyclopedia of Genes and Genomes) pathway enrichment analysis was performed on the differentially expressed proteins. Among the differential proteins, four pathways related to the protein processing in the endoplasmic reticulum, peroxisome, oxidative phosphorylation, and glutathione metabolism were significantly enriched ( P<0.05 ) (Fig. 6).

## 4 Discussion

### 4.1 Effect of resveratrol on morphological indices of horse sperm

During the cryopreservation process of semen, spermatozoa are subject to oxidative stress and apoptosis, leading to decreased sperm motility. Resveratrol (RSV), as a potent antioxidant, can scavenge free radicals and mitigate oxidative stress-induced damage to spermatozoa (Ren et al., 2021; Zhou et al., 2021; Tu et al., 2023). He et al. (2020) found that the addition of 1 mmol L^−1^ RSV to the cryopreservation diluent of porcine semen provided significant protection to post-cryopreservation spermatozoa, significantly reducing oxidative damage incurred during freezing and thawing ( P<0.05 ). However, no notable difference was observed in maintaining sperm motility ( P>0.05 ). In contrast, the findings of this study indicate that, compared to the control group, adding RSV at concentrations of 10, 20, 30, 40, 50, 60, 80, and 100 µ mol L^−1^ to the cryopreservation diluent of equine semen significantly improved the motility of post-cryopreservation spermatozoa ( P<0.05 ). The discrepancy between these two studies may stem from differences in sperm structure between the two species.

The acrosome, a vital structure situated at the head of the spermatozoon, plays an essential role in fertilization by enabling the sperm to penetrate through the oocyte's zona pellucida, thereby facilitating fertilization (Faggi et al., 2023). The assessment of acrosome integrity directly reflects the protective effect on sperm morphology. Bang et al. (2021) discovered that the addition of 200 µ mol L^−1^ RSV to the cryopreservation diluent significantly improved the acrosome integrity rate of dog spermatozoa after cryopreservation and thawing, reaching 58.2 % ( P<0.05 ) compared to 54.6 % in the control group. Our study results demonstrate that the addition of 40 µ mol L^−1^ RSV significantly enhanced the acrosome integrity rate of equine spermatozoa, achieving 74.97 %. The discrepancy in the optimal RSV concentration between these studies and ours may be attributed to physiological characteristics and sperm structural differences among species or may even be influenced by ambient temperature variations. Furthermore, Nogueira et al. (2022) showed that, during semen cryopreservation, RSV effectively maintains sperm acrosome integrity by inhibiting inflammatory responses and reducing sperm cell damage. This protective effect of RSV likely contributed to the significant improvement in the quality of post-cryopreservation spermatozoa of Mongolian horses observed in our study.

The sperm plasma membrane, as the external protective structure, is essential for maintaining sperm morphological stability and ensuring normal physiological function. This membrane not only serves as a barrier but also regulates the interaction between spermatozoa and the external environment, thereby influencing overall biological behavior and fertilization ability (Antonouli et al., 2024). Zhu et al. (2023) found that the addition of RSV to the cryopreservation diluent of sheep resulted in a significant increase in ram sperm plasma membrane integrity with rising RSV concentrations, although this trend was reversed at 100 µ mol L^−1^ ( P<0.05 ). This trend is consistent with our study's findings, suggesting that RSV may mitigate oxidative damage to the sperm cell membrane by scavenging reactive oxygen species (ROSs), thereby preserving membrane integrity. Ahmed et al. (2020) reported that the addition of 50 µ mol L^−1^ RSV to the cryopreservation diluent of buffalo significantly improved plasma membrane integrity, similarly to the optimal concentration observed in sheep. Our study results indicate that the addition of 40 µ mol L^−1^ RSV to the cryopreservation diluent of equine semen significantly enhanced sperm plasma membrane integrity post-cryopreservation ( P<0.05 ), achieving a rate of 55.69 %. The lower optimal RSV concentration (40 µ mol L^−1^) compared to 50 µ mol L^−1^ may be attributed to species-specific differences. Rocha et al. (2017) noted that high RSV concentrations during cryopreservation can significantly alter osmotic pressure, causing spermatozoa to absorb water and swell, potentially leading to the plasma membrane rupturing. Nevertheless, the addition of 40 µ mol L^−1^ RSV in our study likely protected Mongolian horse spermatozoa from osmotic pressure-induced damage, ensuring sperm integrity and motility.

### 4.2 Antioxidant role of resveratrol in the cryopreservation of Mongolian horse semen

During semen cryopreservation, oxidative stress is a non-negligible issue that can lead to an increase in reactive oxygen species (ROSs), subsequently causing oxidative damage to sperm and even leading to sperm death or a sub-lethal state (Chen et al., 2021). Additionally, excessive ROS production may disrupt the integrity of sperm DNA. Total antioxidant capacity (T-AOC) refers to the overall antioxidant level constituted by various antioxidant substances and antioxidant enzymes in biological systems. The primary role of T-AOC is to protect cells and organisms from oxidative stress damage caused by ROS radicals. Research findings by Sun et al. (2020) revealed that adding 150 µ mol L^−1^ of RSV to the boar semen cryopreservation medium significantly enhanced the T-AOC of boar sperm ( P<0.05 ). However, adding 200 µ mol L^−1^ of RSV significantly reduced the T-AOC of sperm ( P<0.05 ), indicating a dose-dependent effect of RSV on sperm T-AOC, which aligns with the trend observed in this study. The results of this experiment found that, within the concentration range of 10–40 µ mol L^−1^, T-AOC gradually increased with the rise in RSV concentration. However, further increasing the RSV concentration led to a gradual decline in T-AOC. Differences in the optimal RSV concentration among different animals may exist, potentially attributable to the structural characteristics of the sperm itself or to variations in experimental methods. Xu et al. (2021a) discovered that, as the RSV concentration increased, the extracellular osmolarity of sperm cells would also increase, leading to sperm dehydration. While this dehydration could effectively prevent the formation of intracellular ice crystals in sperm cells, it might also impact the sperm cell membrane. Water is crucial for maintaining cell membrane stability, and so sperm dehydration could alter the phospholipid bilayer structure of the sperm membrane, thereby reducing sperm antioxidant capacity. From this mechanism, it appears that Mongolian horse sperm is more sensitive to osmolarity changes compared to boar sperm. Zhang et al. (2022) found that adding an appropriate concentration of RSV significantly protected sperm from lipid peroxidation damage and increased the T-AOC of post-frozen sperm ( P<0.05 ). Similar research results have also been verified in the cryopreservation of donkey semen. Therefore, when selecting the RSV concentration for addition, comprehensive consideration of its potential impact on sperm physiological status is necessary.

MDA, as a product of lipid peroxidation, plays a crucial role in assessing the level of oxidative stress (Cordiano et al., 2023). Oxidative stress refers to a phenomenon where the balance between oxidants and antioxidants in the body is disrupted, leading to excessive accumulation of oxidants and subsequent cell damage. Lipid peroxidation, as one of the important markers of cell membrane damage, is closely related to the degree of cell membrane oxidative damage through the generation of its product, MDA (Zhi et al., 2020). When MDA is excessive, it interacts with lipids and proteins, disrupting the structural and functional integrity of the cell membrane and potentially inducing cell apoptosis or necrosis (Zhang et al., 2019). Therefore, quantifying MDA content can indirectly assess the degree of oxidative damage to the cell membrane and further infer the cell's antioxidant capacity. Kaeoket et al. (2023) found that adding 50 µ mol L^−1^ of RSV to the boar cryopreservation medium significantly reduced MDA content ( P<0.05 ), but when the RSV concentration was further increased to 250 µ mol L^−1^, MDA content increased significantly compared to the 50 µ mol L^−1^ group ( P<0.05 ), and its protective effect on sperm was significantly weakened. In this experiment, when 50 µ mol L^−1^ of RSV was added to the horse cryopreservation medium, the MDA content significantly increased compared to the 40 µ mol L^−1^ group. The difference in the optimal RSV concentration may be due to differences between species, as well as the cryopreservation method. It could also be that high RSV concentrations in horse semen cause damage to the sperm cell membrane, leading to excessive ROS production and, ultimately, MDA accumulation.

SOD is an important intracellular antioxidant enzyme whose main function is to scavenge free radicals, particularly converting toxic superoxide anions into hydrogen peroxide and oxygen molecules, thereby mitigating the damage caused by oxygen free radicals to cells and tissues (Kong et al., 2020). Based on different metal cofactors, SOD can be classified into three types: copper-zinc SOD, manganese SOD, and iron SOD, which are distributed in different regions of the cell and are responsible for scavenging superoxide free radicals in their respective regions (Li et al., 2021). This enzyme occupies a central position in the antioxidant defense system and is crucial for alleviating oxidative stress damage to cells and tissues and maintaining organismal health. When the body is exposed to adverse environmental factors (such as radiation, pollution, or tobacco smoke) or internal metabolic processes that generate free radicals, superoxide anions are subsequently generated (Ishihara et al., 2014). At this point, SOD plays a key role by scavenging these toxic superoxide free radicals to maintain cellular redox balance and to protect the body from oxidative stress damage. Therefore, the activity level of SOD can serve as an important indicator for assessing the degree of cellular oxidative damage and antioxidant capacity. Najafi et al. (2019) found that adding RSV to the rooster semen cryopreservation medium did not significantly affect SOD activity ( P>0.05 ), but the results of this experiment showed that adding 10, 20, 30, 40, 50, 60, 80, and 100 µ mol L^−1^ of RSV to the horse semen cryopreservation medium significantly increased SOD activity compared to the control group ( P<0.05 ). The differences in these experimental results may be due to species differences or differences in sperm structure. Shakouri et al. (2021) found that adding RSV to the canine semen cryopreservation medium effectively enhanced the antioxidant capacity of canine sperm, increased SOD activity in sperm, and accelerated the decomposition of oxygen free radicals in cells. Additionally, DHPG (dideoxyhypoxanthine guanine) indirectly eliminates free radicals by inhibiting NADPH (nicotinamide adenine dinucleotide phosphate, reduced form) oxidase activity, thereby enhancing the total antioxidant capacity of sperm. Similarly, adding RSV to the horse cryopreservation medium also significantly increased SOD activity in horse semen. This conclusion is consistent with the findings of Zhu et al. (2019a) and Mortazavi et al. (2020). The ability of RSV to enhance antioxidant capacity within sperm cells may be attributed to its capacity to accelerate the decomposition of oxygen free radicals in sperm and to inhibit the activity of NADPH oxidase, thereby indirectly scavenging free radicals and improving the overall antioxidant capacity of sperm. These research results collectively reveal the potential application value of RSV in protecting sperm from oxidative damage and improving sperm quality.

Despite their minute size, spermatozoa possess an intricate antioxidant system consisting of key components such as GSH, GSH-Px, catalase, taurine, and SOD (Peng et al., 2023). This antioxidant system plays a pivotal role in semen as the first line of defense against lipid peroxidation damage. GSH-Px, a core antioxidant enzyme in this system, eliminates intracellular peroxides by catalyzing the conversion of GSH to oxidized glutathione (GSSG) (Li et al., 2010). Glutathione reductase can then restore GSSG back to GSH using NADPH, thereby maintaining the catalytic activity of GSH-Px. By monitoring NADPH consumption, the activity level of glutathione peroxidase can be accurately assessed (Chang et al., 2022). GSH-Px is widely present in intracellular cells and primarily functions to neutralize free radicals and to protect cells from oxidative stress damage. It converts harmful peroxides into harmless water and alcohol substances, a process crucial for maintaining the intracellular redox balance and protecting the integrity of cell membranes and organelles (Delmonego et al., 2021; Hu et al., 2021; Zhang et al., 2023). When the body is subjected to adverse factors such as oxidative stress, inflammation, or toxins, the expression and activity of GSH-Px increase correspondingly to counter potential oxidative damage (Appiah et al., 2019). Zhu et al. (2019b) found that adding 50 µ mol L^−1^ RSV significantly increased GSH-Px activity levels compared to the control group and other experimental treatment groups ( P<0.05 ). However, further increasing the RSV concentration significantly elevated GSH-Px activity in frozen pig spermatozoa. This is consistent with the findings of the present study, which showed a significant upward trend in GSH-Px activity in spermatozoa as the RSV concentration increased. When the RSV concentration reached 40 µ mol L^−1^, GSH-Px activity was significantly higher than in the control group and in other experimental treatment groups ( P<0.05 ), indicating the strongest antioxidant capacity and the least lipid peroxidation damage to the sperm plasma membrane in this experimental group. Brummer et al. (2013) discovered that adding RSV significantly increased GSH-Px activity levels ( P<0.05 ). This is because RSV promotes the phosphorylation of AMP-activated protein kinase, leading to a reduction in ROS production levels in spermatozoa, thereby decreasing the impact of ROS and reducing damage. The present study may have benefited from this protective effect of RSV, significantly improving the quality of frozen Mongolian horse spermatozoa. This further confirms the superior antioxidant protective effect of RSV.

### 4.3 Differential proteins between the control group and the optimal-resveratrol-concentration group of Mongolian horse spermatozoa

This experiment employed TMT quantitative proteomic sequencing technology to investigate differentially abundant proteins (DAPs) in semen after cryopreservation and thawing between the control group (0 µ mol L^−1^) and the treatment group (40 µ mol L^−1^). The results of DAP analysis revealed that HSPA6, PRDX5, and GPX4 were significantly expressed in the treatment group. These proteins play crucial roles in protecting intracellular proteins from stress-induced damage, promoting proper protein folding, and maintaining normal cellular functioning and antioxidant balance. In contrast, CD47 and PDIA6 were significantly expressed in the control group. HSPA6, a member of the heat shock protein family, was significantly more abundant in the treatment group than in the control group. HSPA6 is primarily localized in the neck region and surface of spermatozoa and regulates sperm survival and apoptosis through interactions with various proteins (Chi et al., 2022). Marín-Briggiler et al. (2010) found that HSPA6 plays a key role in the interaction between spermatozoa and the zona pellucida and promotes sperm capacitation through a calcium-dependent pathway. In this experiment, HSPA6 was found to effectively enhance sperm motility during cryopreservation, which is consistent with previous findings. This protective effect may be attributed to the addition of RSV, which enhances spermatozoa's resistance to stress-induced damage. The peroxiredoxin (PRDX) family, as highly conserved members of the peroxidase family, plays a vital role in maintaining intracellular reactive oxygen species (ROS) homeostasis. Members of this family are widely expressed in various tissues and cells and participate in multiple biological processes, including protecting cells from ROS damage and regulating inflammation, as well as anti-cancer effects, anti-atherosclerosis, and cardiac metabolism. In mammals, six PRDX members have been identified and subdivided into three subfamilies (Lee, 2020). Chen et al. (2020) found that PRDX5 is an antioxidant enzyme present in cells that scavenges oxides and maintains intracellular redox balance. PRDX5 is primarily located in multiple subcellular locations such as the cytoplasm, mitochondria, and nucleus, where it plays an important role in antioxidation and maintaining intracellular redox balance. Consistently with these findings, our results showed that PRDX5 was significantly expressed in the treatment group. GO enrichment analysis revealed that PRDX5 was significantly associated with peroxidase activity. Additionally, KEGG pathway enrichment analysis indicated significant enrichment of PRDX5 in the peroxisome pathway. These findings highlight the important protective role of PRDX5 during sperm cryopreservation, which may be further enhanced by the addition of RSV. GPX4 is a gene in the human genome that encodes the glutathione peroxidase 4 protein (Qian et al., 2023). The glutathione peroxidase family is a class of antioxidant enzymes that play a crucial role in antioxidant protection in cells. GPX4 is a member of this family and primarily participates in regulating the intracellular redox balance (Jia et al., 2020). It catalyzes the reduction of harmful substances such as hydrogen peroxide and organic peroxides, converting them into corresponding alcohols and decomposing them, thereby reducing intracellular oxidative stress and damage. Xing et al. (2023b) reported that GPX4 is present in the mitochondrial structure of spermatozoa. Mitochondria are organelles responsible for energy production in cells and play an important role in providing vitality and energy for spermatozoa. As an antioxidant enzyme, GPX4 helps maintain the redox balance within mitochondria and protects them from oxidative stress damage (Ursini et al., 2020; Xu et al., 2021b; Xie et al., 2023). Del Prete et al. (2019) found that adding RSV to the cryopreservation diluent of equine semen can increase GPX expression levels, inhibit caspase-3 activation, and improve equine sperm motility and membrane integrity. Consistently with these findings, adding 40 µ mol L^−1^ RSV to the cryopreservation diluent of equine semen significantly increased GPX4 expression levels compared to the control group ( P<0.05 ), and this protective effect may be attributed to the addition of RSV. However, the effect of GPX on the cryopreservation of donkey spermatozoa is controversial. Catalán et al. (2022) found that GPX activity levels are negatively correlated with ROS levels in donkey semen, which is consistent with the findings of this study, indicating that increasing GPX protein activity levels can reduce oxidative stress-induced cryopreservation damage to spermatozoa. In this experiment, GPX4 was found to be significantly expressed in the treatment group. GO enrichment analysis showed that GPX4 was significantly associated with phospholipid-hydroperoxide glutathione peroxidase activity. Additionally, the results of KEGG pathway enrichment analysis indicated significant enrichment of GPX4 in the oxidative phosphorylation and glutathione metabolism pathways. These findings further confirm the important protective role of RSV during sperm cryopreservation.

Meanwhile, this study also selected some proteins for WB verification at the protein level. The results demonstrated that the expression levels of HSPA6, PRDX5, and GPX4 in the treatment group were significantly higher than those in the control group ( P<0.05 ), while the expression levels of CD47 in the treatment group were significantly lower than those in the control group ( P<0.05 ). Real-time fluorescent quantitative PCR assays showed that the relative expression levels of *HSPA6*, *PRDX5*, and *GPX4* genes in the treatment group were significantly higher than those in the control group, while the relative expression levels of *PDIA6* and *CD47* genes in the treatment group were significantly lower than those in the control group. Therefore, based on the above analysis, adding RSV not only has significant advantages for sperm cryopreservation but also provides new ideas and feasible optimization schemes for the cryopreservation technology of Mongolian horse semen.

## 5 Conclusions

This study demonstrates the beneficial effects of resveratrol (RSV) on the cryopreservation of horse semen, particularly at a concentration of 40 µ mol L^−1^, which significantly improves sperm quality parameters and antioxidant capacity. RSV's mechanism of action involves modulating key proteins involved in the stress response and redox balance.

## Supplement

10.5194/aab-68-27-2025-supplement The supplement related to this article is available online at: https://doi.org/10.5194/aab-68-27-2025-supplement.

## Data Availability

The data are available from the corresponding author upon request.
