# Supplementary material for: The effect of resveratrol on the cryopreservation of Mongolian horse semen
Source: Arch Anim Breed. 2025 Jan 6;68(1):27–41. doi: 10.5194/aab-68-27-2025 (PMC13225121; doi:10.5194/aab-68-27-2025)
Supplement: The supplement related to this article is available online at: https://doi.org/10.5194/aab-68-27-2025-supplement. [file aab-68-27-2025-supplement.zip › aab-68-27-2025-supplement-title-page.pdf]

Supplement of Arch. Anim. Breed., 68, 27–41, 2025
https://doi.org/10.5194/aab-68-27-2025-supplement
© Author(s) 2025. CC BY 4.0 License.

Open Access
Archives Animal Breeding

Supplement of

The effect of resveratrol on the cryopreservation of Mongolian horse semen

Ming Du et al.

Correspondence to: Manglai Dugarjaviin (dmanglai@163.com)

- aab-68-27-2025-supplement-title-page.pdf
- Table S1.xlsx
- Table S2.xlsx
- Table S3.xlsx
- Table S4.xlsx

The copyright of individual parts of the supplement might differ from the article licence.
